# Serum glycated hemoglobin level as a predictor of atrial fibrillation: A systematic review with meta-analysis and meta-regression

**DOI:** 10.1371/journal.pone.0170955

**Published:** 2017-03-07

**Authors:** Wenwei Qi, Nixiao Zhang, Panagiotis Korantzopoulos, Konstantinos P. Letsas, Min Cheng, Fusheng Di, Gary Tse, Tong Liu, Guangping Li

**Affiliations:** 1 Tianjin Key Laboratory of Ionic-Molecular Function of Cardiovascular disease, Department of Cardiology, Tianjin Institute of Cardiology, Second Hospital of Tianjin Medical University, Tianjin, China; 2 School of Public Health, Chinese Academy of Medical Sciences and Peking Union Medical College, Beijing, China; 3 First Department of Cardiology, University of Ioannina Medical School, Ioannina, Greece; 4 Second Department of Cardiology, Laboratory of Cardiac Electrophysiology, “Evangelismos” General Hospital of Athens, Athens, Greece; 5 State Key Laboratory of Cardiovascular Disease, Fuwai Hospital, National Center for Cardiovascular Diseases, Chinese Academy of Medical Sciences and Peking Union Medical College, Beijing, China; 6 Department of Endocrinology and Metabolism, the Third Central Hospital of Tianjin, Tianjin, China; 7 Department of Medicine and Therapeutics, Chinese University of Hong Kong, Hong Kong, Special Administrative Region, P.R. China; 8 Li Ka Shing Institute of Health Sciences, Chinese University of Hong Kong, Hong Kong, Special Administrative Region, P.R. China; Scuola Superiore Sant'Anna, ITALY

## Abstract

**Background and Aim:**

Glycated hemoglobin (HbA1c) is a long-term measure of glucose control. Although recent studies demonstrated a potential association between HbA1c levels and the risk of atrial fibrillation (AF), the results have been inconsistent. The aim of this meta-analysis is to evaluate the utility of HbA1c level in predicting AF.

**Methods:**

PubMed and the Cochrane Library databases were searched for relevant studies up to March 2016. Prospective cohort studies and retrospective case-control studies were included. Relative risk (RR) or odds ratio (OR) with 95% confidence intervals (CIs) of AF development were determined for different HbA1c levels. The random effect model was conducted according to the test of heterogeneity among studies. Subgroup analyses and meta-regression models were carried out to identify potential sources of heterogeneity.

**Results:**

Eight prospective cohort studies with 102,006 participants and 6 retrospective case-control studies with 57,669 patients were finally included in the meta-analysis. In the primary meta-analysis, HbA1c levels were not associated with an increased risk of AF whether as a continuous (RR, 1.06; 95% CI, 0.96–1.18) or categorical variable (RR, 0.99; 95% CI, 0.83–1.18). Nevertheless, prospective studies showed about 10% increased risk of AF with elevated HbA1c levels both as a continuous (RR, 1.11; 95% CI, 1.06–1.16) and as a categorical variable (RR, 1.09; 95% CI, 1.00–1.18). In subgroup analyses, pooled results from studies with longer follow-up durations, published after 2012, aged < 63 years, with exclusion of cardiac surgery patients demonstrated an increased risk of AF for every 1% increase in HbA1c levels, while studies conducted in the United States with longer follow-up (more than 96 months), larger sample size and higher quality score (≥6) showed an increased risk of AF for higher HbA1c level as a categorical variable.

**Conclusions:**

Elevated serum HbA1c levels may be associated with an increased risk of AF, but further data are needed. Serum HbA1c levels might be considered as a potential biomarker for prediction of AF.

## Introduction

Atrial fibrillation (AF) is the most common arrhythmia occurring in 2.3–3.4% of the general population and its prevalence is estimated to be at least doubled by 2050 [1]. AF represents a major public health problem with a significant impact on cardiovascular morbidity and mortality as well as on health care cost [2,3]. Glycated hemoglobin (HbA1c) is a reliable biochemical marker of glucose control over the preceding 2–3 months and is widely used in daily clinical practice. The association between HbA1c and AF has been investigated in previous studies but the findings have been controversial [3–7]. A recent study from Japan showed that higher HbA1c is associated with a decreased risk of AF [8], while the Atherosclerosis Risk in Communities Study showed an increased risk of AF [9]. Therefore, we performed a comprehensive meta-analysis to evaluate the current evidence regarding the potential association between HbA1c and AF risk.

## Materials and methods

The Preferred Reporting Items for Systematic Reviews and Meta-Analysis (PRISMA) statement [10] was used in this study (detailed in S1 PRISMA Checklist).

### Literature search strategy and study selection

A literature search of Pubmed and Cochrane Library was performed using the key words (“hemoglobin A, glycosylated” or “glycated hemoglobin” or “hemoglobin A1c” or “HbA1c” or “HgbA1c”) and (“atrial fibrillation”) from inception to March 2016, with English language restriction. The search was limited to studies carried out in humans only. We reviewed all articles with an abstract suggesting relevance and we checked the reference lists of all relevant articles to identify other eligible studies. Both prospective studies and retrospective studies were included in this meta-analysis. Studies which reported glucose without HbA1c or other types of arrhythmias but no AF (or flutter) were excluded.

### Data extraction and quality evaluation

The following details were recorded for each study: author, publication year, study design, country of origin, study population, study period/duration of the follow-up, number of subjects, number of events, sex, age, health at baseline, odds ratios (ORs), relative risks (RRs) or hazard ratios (HRs) with corresponding 95% confidence intervals (CIs) for HbA1c, and confounding factors adjusted in multivariable analysis. Literature search, data extraction and study quality evaluation were conducted independently by two investigators (W.Q. and N.Z.), with disagreements resolved by a third investigator (T.L.). The Newcastle-Ottawa (Quality Assessment) Scale (NOS) was used to assess bias in included studies.

### Statistical analysis

In this meta-analysis, the adjusted RRs with 95% CIs were considered as the effect size for all studies, while HRs and ORs were directly deemed equivalent to RRs because of the incidence of AF is rare [11]. As the studies included in this meta-analysis used HbA1c level as either a continuous or categorical variable, two separate meta-analyses were performed for both types of variables to evaluate the association between HbA1c levels and AF occurrence. The level of HbA1c was combined into dichotomous variable if there are more than two categories of HbA1c in the studies with HbA1c levels as the categorical variable. Estimates of higher level of HbA1c were pooled with the lower quintile to derive an overall RR, which was subsequently used for pooled analysis.

Weighted random effects model for pooling effect sizes was used. I^2^ and H^2^ statistic were used to test heterogeneity among studies [12]. To determine the robustness of the combined estimates, subgroup analyses by country (America vs. not America), year of publication (before 2012 vs. after 2012), sample size (<3000 participants vs. ≥ 3000 participants), duration of follow-up (< 96 months vs. ≥ 96 months), age (<63 years vs.≥ 63 years), quintiles (2 vs. 3 vs. 4), prime effect size (OR vs. RR vs. HR), study quality (quality score ≤ 6 vs. quality score > 6), and coronary artery bypass grafting (CABG, yes vs. no) were performed. Meta-regression analysis was carried out to identify potential sources of heterogeneity between studies.

Sensitivity analyses excluding those studies in which crude RRs were calculated or had the least number of enrolled patients were performed. Publication bias was assessed using the funnel plots, Begg’s and Egger’s adjusted rank correlation test. All statistical analyses were conducted with STATA 11 (STATA Corp, College Station, Texas, USA). All reported probabilities were 2-sided with P < 0.05 considered statistically significant.

## Results

### Literature search

**Fig 1:**
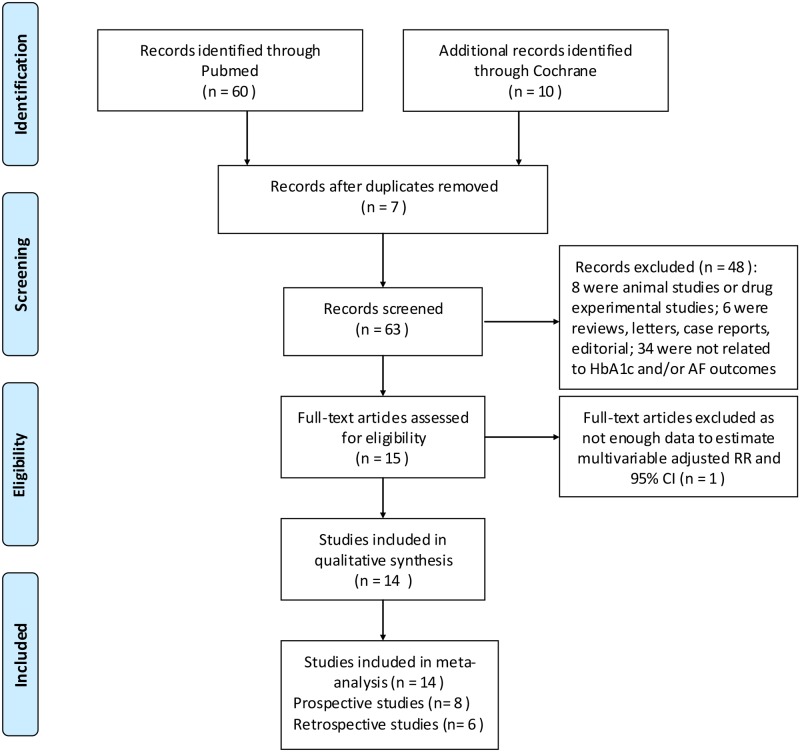
Flow chart of selection of studies for inclusion in meta-analysis. *From*: Moher D, Liberati A, Tetzlaff J, Altman DG, The PRISMA Group (2009). *P*referred *R*eporting *I*tems for *S*ystematic Reviews and *M*eta-*A*nalyses: The PRISMA Statement. PLoS Med 6(6): e1000097. doi:10.1371/journal.pmed1000097.

A flow diagram depicting the search and selection process is shown in the Fig 1. 63 citations were retrieved on the initial search. After removal of 49 citations at the title, abstract level, or full-text assessment, 14 articles were included in our pooled analyses. Specifically, 8 prospective studies and 6 retrospective studies were analyzed.

### Study characteristics

Baseline characteristics of the 14 included studies are shown in Table 1. These studies included 159,675 participants and 6,340 cases of interest, while the number of participants per study ranged from 101 to 52,448. Of the 14 studies, 6 were conducted in United States, 3 in Japan, 1 in China, 1 in Ireland, 1 in Turkey, 1 in Spain and 1 in 40 countries. Eight studies were published after 2012, while 6 studies were published between 2006 and 2011. Twelve studies recruited both male and female participants, while 2 studies involved only female subjects. Study subjects were aged between 38 and 89 years at baseline with a mean age of 63 years. The follow-up durations of prospective studies ranged from 6 months to 16.4 years and the median was 96 months. Possible confounding factors were adjusted in 10 studies, 7 of which adjusted for age. The mean overall quality score of the studies was 6, with a range of 3–9, and 9 studies had quality scores greater than or equal to 6.

**Table 1:** Characteristics of prospective and retrospective studies on HbA1c levels and risk of atrial fibrillation.

author	Publication year	country	study population	study period(follow-up duration)	participants, N	Events, N	female,%	age, mean(range), years	RR(95%CI)	quality score
Prospective studies										
Latini, R	2013	40 countries	White, Black, Asian	mean 6.5 y	8943	613	51.30%	63	1.10(0.91,1.32)^a^	7
Z.-H. Lu	2014	China	symptomatic PAF with T2DM	2012–2013 (mean 1y)	149	50	57.72%	62	1.22(1.02,1.47)^a^	6
Rachel R. Huxley	2012	USA	whites and African-Americans	1987–2007 (mean 14.5 y)	13025	1311	55.90%	57	1.11(1.05,1.16)^a^	8
Tobias Schoen	2012	USA	female health professionals	mean 16.4 y	34720	835	100%	53(≥45)	1.06(0.94,1.19)^a^	8
Roopinder K. Sandhu	2014	USA	female health professionals	mean 16.4 y	34720	1039	100%	53(≥45)	1.05(0.86,1.28)^a^	8
Kaoru Matsuura	2009	Japan	diabetic patients who had undergone OPCAB	2000–2007 (mean2.2y)	101	26	20.79%	65	1.49(0.61,3.64)^b^	3
Omid Fatemi	2014	USA, Canada	volunteers with DM	mean 4.68 y	10,082	159	38%	62(40–79)	0.92(0.67,1.25)^b^	5
C.J. O’Sullivan	2006	Galway,Ireland	people were admitted for emergency and elective vascular surgical procedures	mean 0.5y	165	5	41%	72(48–88)	0.21(0.02,1.86)^b^	3
retrospective studies										
Yasuyuki Iguchi	2010	Japan	Japanese adults in Kurashiki-city companies, offices, government or factories.	2006–2007	52448	1161	65.72%	72(65–78)	1.18(1.09,1.28)^c^	7
Takeshi Kinoshita	2011	Japan	patients underwent CABG	2002–2010	805	159	20.25%	68	0.74(0.60,0.92)^c^	6
Michael E. Halkos	2008	USA	patients underwent CABG	2002–2006	3089	549	27.39%	63	0.89(0.80,0.98)^c^	6
Turgut, O	2013	Turkey	diabetic patients admitted to cardiology and endocrinology outpatient clinics for routine examination	2011.1–5	162	81	48.15%	63(38–89)	1.87(0.75,3.01)^c^	5
Maria L Blasco	2014	Spain	patients with AMI and unknown diabetes mellitus	2009–2013	601	73.00	22%	62	0.37(0.20,0.67)^d^	4
Sascha Dublin	2010	USA	Treated diabetes	mean 8.2 years	564	253	25.21%	70	1.14(0.96,1.35)^c^	9

^a^Hazard Ratio from the original literature.

^b^Relative Risk generated from the number of atrial fibrillation occurrence in different HbA1c levels.

^c^Odds Ratio from the original literature.

^d^Odds Ratio generated from the number of atrial fibrillation occurrence in different HbA1c levels.

### HbA1c and atrial fibrillation

Of the 14 selected studies, 3 studies [4,9,13] reported that higher HbA1c levels increase the risk of AF, 3 studies [7,8,14] demonstrated that higher HbA1c levels are a protective factor for AF, and the remaining eight studies [5,6,11,15–19] showed no significant association between HbA1c levels and AF.

**Fig 2:**
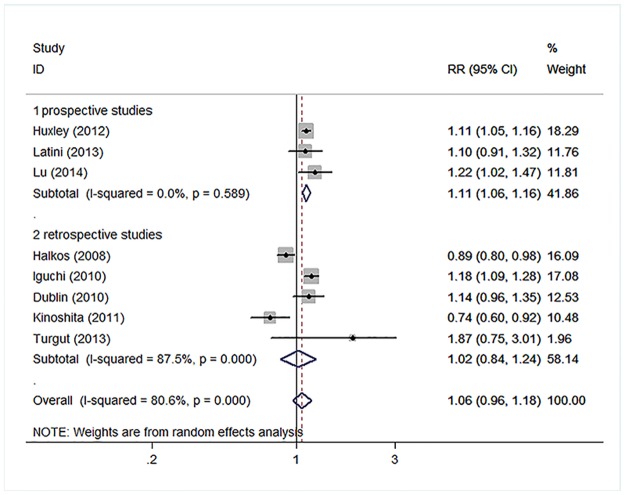
Forest plot demonstrating the association between HbA1c levels and AF depending on different study styles which HbA1c levels were analyzed as continuous variable. Relative risks of AF for higher HbA1c compared with lower HbA1c. Squares indicate study-specific risk estimates (size of the square reflects the study-specific statistical weight, that is, the inverse of the variance); horizontal lines indicate 95% CIs; diamond indicates summary risk estimate with its corresponding 95% CI.

**Fig 3:**
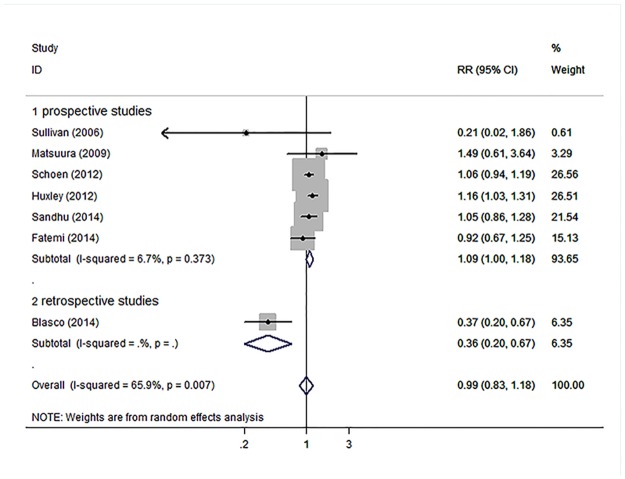
Forest plot demonstrating the association between HbA1c levels and AF depending on different study styles which HbA1c levels were analyzed as categorical variable. Relative risks of AF for higher HbA1c compared with lower HbA1c. Squares indicate study-specific risk estimates (size of the square reflects the study-specific statistical weight, that is, the inverse of the variance); horizontal lines indicate 95% CIs; diamond indicates summary risk estimate with its corresponding 95% CI.

In the primary meta-analysis, HbA1c levels were not associated with an increased risk for AF both as a continuous variable (RR, 1.06; 95% CI, 0.96–1.18) with significant heterogeneity (H^2^ = 5.15 and I^2^, 80.6%; 95% CI, 62.5%-89.9%; Fig 2) as well as a categorical variable (RR, 0.99; 95% CI, 0.83–1.18) with significant heterogeneity (H^2^ = 2.93 and I^2^, 65.9%; 95% CI, 23.6%-84.8%; Fig 3). However, prospective studies showed nearly 10% increased risk of AF with increased HbA1c levels, both as a continuous variable (RR, 1.11; 95% CI, 1.06–1.16) without significant heterogeneity (H^2^ = 0.53 and I^2^, 0.0%; 95% CI, 0.0%-89.6%; Fig 2) and as a categorical variable (RR, 1.09; 95% CI, 1.00–1.18) without significant heterogeneity (H^2^ = 1.07 and I^2^, 6.7%; 95% CI, 0.0%-76.3%; Fig 3). We also performed analyses among diabetic patients and non-diabetic patients, but there was not statistically significant association between HbA1c and AF. The pooled RR for higher HbA1c levels compared with lower HbA1c levels was 1.09 (95% CI: 0.79, 1.51) and 0.82 (95% CI: 0.43, 1.55) among diabetic and non-diabetic patients, respectively.

Relative risks of AF for higher HbA1c compared with lower HbA1c. Squares indicate study-specific risk estimates (size of the square reflects the study-specific statistical weight, that is, the inverse of the variance); horizontal lines indicate 95% CIs; diamond indicates summary risk estimate with its corresponding 95% CI.

Relative risks of AF for higher HbA1c compared with lower HbA1c. Squares indicate study-specific risk estimates (size of the square reflects the study-specific statistical weight, that is, the inverse of the variance); horizontal lines indicate 95% CIs; diamond indicates summary risk estimate with its corresponding 95% CI.

### Subgroup and sensitivity analyses

Subgroup, meta-regression and sensitivity analyses were performed to identify the origin of heterogeneity in the studies included.

To explore the heterogeneity among these studies, subgroup analyses were performed based on a number of key study characteristics. When analyzing studies using the HbA1c levels as a continuous variable, the effects of higher HbA1c on AF varied significantly across study design, publication year, follow-up duration, age and CABG (Table 2). Increased AF risk was observed in patients with higher HbA1c levels in studies which i) were prospective, ii) were published after 2012, iii) had longer follow-up duration, iv) included subjects aged < 63 years, v) concerned with prime effect size HR, and vi) excluded post-CABG AF. In addition, no heterogeneity was evident in these subgroup analyses (P > 0.05). We also performed subgroup analyses for several key study characteristics among studies using the HbA1c levels as a categorical variable. We demonstrated that higher HbA1c predicted the development of AF in prospective studies, in those conducted in the United States, in studies with longer follow-up (more than 96 months), and in those with larger sample size, in those with prime effect size HR, and in studies with higher quality score (≥6) (Table 3).

**Table 2:** Stratified analyses of pooled relative risks (95% CI) of AF for HbA1c levels as continuous variable.

Stratified analyses	No. of studies	Pooled RR(95% CI)	Heterogeneity test	
			P value	I^2^(%)
style				
prospective	3	**1.11(1.06,1.16)**	0.59	0.00%
retrospective	5	1.02(0.84,1.24)	<0.01	87.50%
Country				
America	3	1.04(0.89,1.21)	<0.01	86.60%
Not America	4	1.09(0.85,1.41)	<0.01	84.10%
Publication year				
before 2011	4	0.98(0.80,1.20)	<0.01	89.80%
After 2012	4	**1.20(1.06,1.19)**	0.36	5.70%
Sample size				
<3000	4	1.09(0.82,1.44)	<0.01	81.70%
≥3000>ze7H耂 ≥96m>ez7I耂 ≥6>ez71.13(1.09,1.18)J耀 1.13(1.09,1.18)70.	4	1.06(0.96,1.18)	<0.01	84.60%
Follow-up duration				
<96m	2	**1.16(1.02,1.32)**	0.43	0.00%
≥96m>ez7I耂 ≥6>ez71.13(1.09,1.18)J耀 1.13(1.09,1.18)70.83(	1	**1.11(1.05,1.16)**	.	.
age				
<63y	2	**1.12(1.06,1.18)**	0.31	4.30%
≥63y	6	1.03(0.88,1.22)	<0.01	84.60%
prime effect size				
OR	5	1.02(0.84,1.24)	<0.01	87.50%
RR	0	.	.	.
HR	3	**1.11(1.06,1.16)**	0.59	0.00%
CABG				
Yes	2	**0.83(0.70,0.99)**	0.13	57.20%
No	6	**1.13(1.09,1.18)**	0.46	0.00%
Study quality				
<6	1	1.87(0.93,3.76)	.	.
≥6>ez71.13(1.09,1.18)J耀 1.13(1.09,1.18)70.83(0.70,0.99)K耀0.83(0	7	1.05(0.95,1.16)	<0.01	82.20%

Note: I^2^ is interpreted as the proportion of total variation across studies that is due to heterogeneity rather than chance; CABG, coronary artery bypass grafting.

**Table 3:** Stratified analyses of pooled relative risks (95% CI) of AF for HbA1c levels as categorical variable.

Stratified analyses	No. of studies	Pooled RR(95% CI)	Heterogeneity test	
			P value	I^2^(%)
style				
prospective	6	**1.09(1.00,1.18)**	0.37	6.70%
retrospective	1	**0.37(0.20,0.67)**	.	.
Country				
America	4	**1.09(1.01,1.17)**	0.44	0.00%
Not America	3	0.57(0.18,1.77)	0.03	72.30%
Publication year				
before 2011	2	0.73(0.12,4.59)	0.10	62.20%
After 2012	5	0.99(0.83,1.18)	<0.01	73.20%
Sample size				
<3000	3	0.57(0.18,1.77)	0.03	72.30%
≥3000>ze7H耂 ≥96m>ez7I耂 ≥6>ez71.13(1.09,1.18)J耀1.13(1.09,1.18)70.	4	**1.09(1.01,1.17)**	0.44	0.00%
Follow-up duration				
<96m	3	0.95(0.56,1.63)	0.24	29.70%
≥96m>ez7I耂 ≥6>ez71.13(1.09,1.18)J耀 1.13(1.09,1.18)70.83(	3	**1.10(1.02,1.19)**	0.48	0.00%
age				
<63y	5	0.99(0.83,1.18)	<0.01	73.20%
≥63y	2	0.73(0.12,4.59)	0.10	62.20%
quintiles				
2	3	0.95(0.56,1.63)	0.24	29.70%
3	2	0.68(0.22,2.11)	<0.01	92.60%
4	2	1.05(0.95,1.17)	0.96	0.00%
prime effect size				
OR	1	**0.37(0.20,0.67)**	.	.
RR	3	0.95(0.56,1.63)	0.24	29.70%
HR	3	**1.10(1.02,1.19)**	0.48	0.00%
Adjustment for CABG				
Yes	1	1.49(0.61,3.64)	.	.
No	6	0.98(0.82,1.17)	<0.01	70.70%
Study quality				
<6	4	0.69(0.35,1.36)	0.02	71.00%
≥6	3	**1.10(1.02,1.19)**	0.48	0.00%

Note: I^2^ is interpreted as the proportion of total variation across studies that is due to heterogeneity rather than chance; CABG, coronary artery bypass grafting.

In the meta-regression analysis of all 14 studies, the influence of study design, country of origin, publication year, sample size, CABG setting, and study quality on heterogeneity were explored. None of these parameters contributed significantly to substantial heterogeneity (P > 0.05).

We further performed sensitivity analyses as follows. Of the 8 studies that analyzed HbA1c levels as continuous variable, and after excluding the study by Turgut [16], which had the least number of enrolled patients, sensitivity analysis did not reveal significant influence on overall results (RR, 1.05; 95% CI, 0.95–1.16); with significant heterogeneity (I^2^, 82.2%; 95% CI, 64.4%-91.1%). Of the 7 studies using HbA1c level as categorical variable, only 3 studies reported multivariable adjusted RRs on AF for higher HbA1c compared with lower HbA1c levels. After removing the study without multivariable adjusted RRs, significant difference was not found in the heterogeneity among the remaining 3 studies (I^2^, 0.0%; 95% CI, 0.0%-89.6%) and the results were the same as the prospective cohort studies (RR, 1.10; 95% CI, 1.02–1.19).

### Publication bias

**Fig 4:**
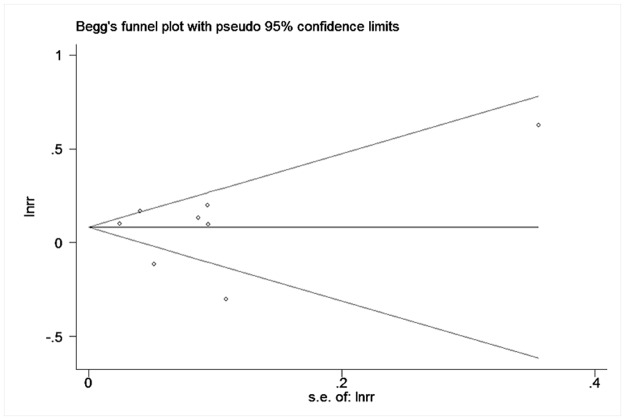
Funnel plots of continuous variable results included in meta-analysis.

**Fig 5:**
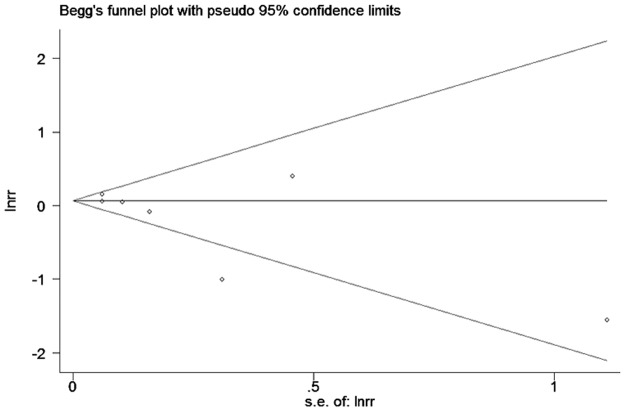
Funnel plots of categorical variable results included in meta-analysis.

Neither funnel plots (Figs 4 and 5) nor Egger’s and Begg’s tests revealed evidence of publication bias (p = 0.81 for Begg’s test and p = 0.80 for Egger’s test in the studies using HbA1c levels as continuous variable; p = 0.29 with Begg’s test and p = 0.13 with Egger’s test in the studies using HbA1c levels as categorical variable).

## Discussion

In this comprehensive meta-analysis, we demonstrated that increased HbA1c levels were associated with an increased risk of AF in prospective cohort studies but not in case-control studies. A recent study from Eastern Norway indicated a significant positive correlation between HbA1c levels and duration of AF (r = 0.408, p = 0.005), thereby implicating abnormal glucose metabolism in increasing AF burden [20]. Given that diabetes increases thromboembolic risk in AF and further promotes the arrhythmia, HbA1c levels could be a useful marker in strategies aiming to reduce AF burden and its related complications. In support of this notion, Saliba et al. recently showed that glycated hemoglobin is directly associated with stroke risk, and the use of HbA1c improved accuracy for predicting stroke events in diabetic patients with AF [21]. Based on retrospective data with the effect size of standardized mean difference (SMDs) mostly among Chinese subjects, Yang et al. demonstrated elevated HbA1c levels could increase the risk of AF in patients with diabetes mellitus (DM) [22]. We further investigated this in the general population with the effect size of RRs, both based on prospective and retrospective studies conducted globally.

We also performed subgroup analyses examining potentially significant factors such as publication year, sample size, age, geographical origin, and study quality that may affect the authenticity of the potential association between HbA1c levels and AF development. Thus, we showed that studies conducted in the United States, studies published after 2012, those with longer follow-up duration, studies with larger sample size, those with higher quality score and those not examining postoperative AF had significant influence on our results, implying that these factors may be the source of the heterogeneity.

Poor glycemic control reflected by increased HbA1c levels is an independent risk factor of AF [23]. Several underlying pathophysiological mechanisms have been proposed to explain this association. Firstly, as a long half-life protein, HbA1c has been suggested as reliable tool not only for diagnosing DM but also for identifying individuals at increased risk for cardiovascular events whether or not DM is present [24,25]. High levels of HbA1c were associated not only with the long-term disorder of glycolipid metabolism but also with low-grade systematic inflammation and the progression of atherosclerotic disease [26]. In a population study, high HbA1c levels have been associated with biomarkers of systemic inflammation such as C-reactive protein (CRP), D-dimer, uric acid, white blood cell count and fibrinogen [27]. Of note, inflammation and oxidative stress have been associated with AF development [28]. Moreover, overexpression of collagen proteins might contribute to the prolongation of the atrial activation time and cycle length, and to the reduction of atrial voltage, which create a substrate for the development and perpetuation of AF [29]. Therefore, increased HbA1c levels might be involved in the inflammatory state implicated in AF pathophysiology.

### Study limitations

We acknowledge several limitations of our study. Firstly, despite the lack of indication of major publication bias in the formal evaluations employed, potential publication bias cannot be completely excluded. This may be responsible for some heterogeneity and underpowered meta-regression, especially in the light of the small number of studies. Secondly, since we focused on the serum levels of HbA1c more frequently, a subset of well-controlled diabetic patients are at an increased risk of AF could be misclassified into the low serum levels group, especially in case-control studies. Besides, when the level of HbA1c was analyzed as a categorical variable, the RRs were combined into dichotomous variable if more than two groups of RRs were present. Moreover, the thresholds of HbA1c levels were different across the included studies. Nevertheless, no significant difference was found among different quintiles of RRs and the cutoff points were similar between 5.7% to 7.0%. In addition, both retrospective and prospective studies were pooled together and different statistical parameters like RR, OR, HR were treated as equivalent for analysis purposes. The differences among different prime effect sizes may be due to study style. Some AF patients did not visit hospital regularly and some AF cases might have been undetected because many AF episodes were asymptomatic, which might underestimate of AF. Indeed, the only way to obtain a true picture of AF prevalence would be the use of long-term ECG or Holter monitoring.

## Conclusions

This comprehensive meta-analysis suggests that elevated serum HbA1c levels were associated with an increased risk of AF in prospective studies, and therefore serum HbA1c levels may be viewed as a potential biomarker to predict AF and as a tool for AF prevention. Undoubtedly, further prospective studies with larger population sizes are needed to elucidate the exact prognostic role of HbA1c in AF development. Finally, the use of HbA1c levels as a prognostic and monitoring tool in the management of AF as well as its related complications should be evaluated carefully.

## Supporting information

S1 PRISMA Checklist PRISMA checklist. (DOC)

## References

[1] Ball J, Carrington MJ, McMurray JJ, Stewart S. Atrial fibrillation: profile and burden of an evolving epidemic in the 21st century. INT J CARDIOL. 2013;167(5):1807–24. 10.1016/j.ijcard.2012.12.093 23380698

[2] Camm AJ, Kirchhof P, Lip GY, Schotten U, Savelieva I, Ernst S et al Guidelines for the management of atrial fibrillation: the Task Force for the Management of Atrial Fibrillation of the European Society of Cardiology (ESC). EUROPACE. 2010;12(10):1360–420. 10.1093/europace/euq350 20876603

[3] Fuster V, Ryden LE, Cannom DS, Crijns HJ, Curtis AB, Ellenbogen KA et al 2011 ACCF/AHA/HRS focused updates incorporated into the ACC/AHA/ESC 2006 guidelines for the management of patients with atrial fibrillation: a report of the American College of Cardiology Foundation/American Heart Association Task Force on practice guidelines. CIRCULATION. 2011;123(10):e269–367. 10.1161/CIR.0b013e318214876d 21382897

[4] Lu ZH, Liu N, Bai R, Yao Y, Li SN, Yu RH et al HbA1c levels as predictors of ablation outcome in type 2 diabetes mellitus and paroxysmal atrial fibrillation. HERZ. 2015;40 Suppl 2:130–6. 25336239 10.1007/s00059-014-4154-6

[5] Fatemi O, Yuriditsky E, Tsioufis C, Tsachris D, Morgan T, Basile J et al Impact of intensive glycemic control on the incidence of atrial fibrillation and associated cardiovascular outcomes in patients with type 2 diabetes mellitus (from the Action to Control Cardiovascular Risk in Diabetes Study). AM J CARDIOL. 2014;114(8):1217–22. 10.1016/j.amjcard.2014.07.045 25159234 PMC4291278

[6] Sandhu RK, Conen D, Tedrow UB, Fitzgerald KC, Pradhan AD, Ridker PM et al Predisposing factors associated with development of persistent compared with paroxysmal atrial fibrillation. J AM HEART ASSOC. 2014;3(3):e916. 10.1161/JAHA.114.000916 PMC4309092 24786144

[7] Blasco ML, Sanjuan R, Palacios L, Huerta R, Carratala A, Nunez J et al Prognostic value of admission glycated haemoglobin in unknown diabetic patients with acute myocardial infarction. Eur Heart J Acute Cardiovasc Care. 2014;3(4):347–53. 10.1177/2048872614530574 24676027

[8] Kinoshita T, Asai T, Suzuki T, Kambara A, Matsubayashi K. Preoperative hemoglobin A1c predicts atrial fibrillation after off-pump coronary bypass surgery. Eur J Cardiothorac Surg. 2012;41(1):102–7. 10.1016/j.ejcts.2011.04.011 21612941 PMC3241086

[9] Huxley RR, Alonso A, Lopez FL, Filion KB, Agarwal SK, Loehr LR et al Type 2 diabetes, glucose homeostasis and incident atrial fibrillation: the Atherosclerosis Risk in Communities study. HEART. 2012;98(2):133–8. 10.1136/heartjnl-2011-300503 21930722 PMC3237721

[10] Moher D, Liberati A, Tetzlaff J, Altman DG. Preferred reporting items for systematic reviews and meta-analyses: the PRISMA statement. PLOS MED. 2009;6(7):e1000097 10.1371/journal.pmed.1000097 19621072 PMC2707599

[11] Latini R, Staszewsky L, Sun JL, Bethel MA, Disertori M, Haffner SM et al Incidence of atrial fibrillation in a population with impaired glucose tolerance: the contribution of glucose metabolism and other risk factors. A post hoc analysis of the Nateglinide and Valsartan in Impaired Glucose Tolerance Outcomes Research trial. AM HEART J. 2013;166(5):935–40. 10.1016/j.ahj.2013.08.012 24176451

[12] Ioannidis J, Patsopoulos N, Evangelou E. Uncertainty in heterogeneity estimates in meta-analyses.*BMJ*.2007;335(7626):914–916. 10.1136/bmj.39343.408449.80 17974687 PMC2048840

[13] Iguchi Y, Kimura K, Shibazaki K, Aoki J, Sakai K, Sakamoto Y et al HbA1c and atrial fibrillation: a cross-sectional study in Japan. INT J CARDIOL. 2012;156(2):156–9. 10.1016/j.ijcard.2010.10.039 21093939

[14] Halkos ME, Puskas JD, Lattouf OM, Kilgo P, Kerendi F, Song HK et al Elevated preoperative hemoglobin A1c level is predictive of adverse events after coronary artery bypass surgery. J Thorac Cardiovasc Surg. 2008;136(3):631–40. 10.1016/j.jtcvs.2008.02.091 18805264

[15] Dublin S, Glazer NL, Smith NL, Psaty BM, Lumley T, Wiggins KL et al Diabetes mellitus, glycemic control, and risk of atrial fibrillation. J GEN INTERN MED. 2010;25(8):853–8. 10.1007/s11606-010-1340-y 20405332 PMC2896589

[16] Turgut O, Zorlu A, Kilicli F, Cinar Z, Yucel H, Tandogan I et al Atrial fibrillation is associated with increased mean platelet volume in patients with type 2 diabetes mellitus. PLATELETS. 2013;24(6):493–7. 10.3109/09537104.2012.725876 22994845

[17] O'Sullivan CJ, Hynes N, Mahendran B, Andrews EJ, Avalos G, Tawfik S et al Haemoglobin A1c (HbA1C) in non-diabetic and diabetic vascular patients. Is HbA1C an independent risk factor and predictor of adverse outcome? Eur J Vasc Endovasc Surg. 2006;32(2):188–97. 10.1016/j.ejvs.2006.01.011 16580235

[18] Matsuura K, Imamaki M, Ishida A, Shimura H, Niitsuma Y, Miyazaki M. Off-pump coronary artery bypass grafting for poorly controlled diabetic patients. Ann Thorac Cardiovasc Surg. 2009;15(1):18–22 19262445

[19] Schoen T, Pradhan AD, Albert CM, Conen D. Type 2 diabetes mellitus and risk of incident atrial fibrillation in women. J AM COLL CARDIOL. 2012;60(15):1421–8. 10.1016/j.jacc.2012.06.030 22981550 PMC4277997

[20] Johansen OE, Brustad E, Enger S, Tveit A. Prevalence of abnormal glucose metabolism in atrial fibrillation: a case control study in 75-year old subjects. CARDIOVASC DIABETOL. 2008;7:28 10.1186/1475-2840-7-28 18822173 PMC2564913

[21] Saliba W, Barnett-Griness O, Elias M, Rennert G. Glycated Hemoglobin and Risk of First Episode Stroke in Diabetic Patients with Atrial Fibrillation: A Cohort Study. HEART RHYTHM. 2015. 10.1016/j.hrthm.2015.01.025 25614249

[22] Yang YF, Zhu WQ, Cheng K, Chen QX, Xu Y, Pang Y et al Elevated glycated hemoglobin levels may increase the risk of atrial fibrillation in patients with diabetes mellitus. INT J CLIN EXP MED. 2015;8(3):3271–80 26064216 PMC4443050

[23] Goudis CA, Korantzopoulos P, Ntalas IV, Kallergis EM, Liu T, Ketikoglou DG. Diabetes mellitus and atrial fibrillation: Pathophysiological mechanisms and potential upstream therapies. INT J CARDIOL. 2015;184:617–22. 10.1016/j.ijcard.2015.03.052 25770841

[24] Sacks DB, Arnold M, Bakris GL, Bruns DE, Horvath AR, Kirkman MS et al Guidelines and recommendations for laboratory analysis in the diagnosis and management of diabetes mellitus. DIABETES CARE. 2011;34(6):e61–99. 10.2337/dc11-9998 21617108 PMC3114322

[25] Zhao X, Chang MH, Chen L, Jiang L, He M, Chen J et al An increased level of haemoglobin A1C predicts a poorer clinical outcome in patients with acute pancreatitis. Clin Endocrinol (Oxf). 2012;77(2):241–5. 21988175 10.1111/j.1365-2265.2011.04252.x

[26] Daida H, Takayama T, Hiro T, Yamagishi M, Hirayama A, Saito S et al High HbA1c levels correlate with reduced plaque regression during statin treatment in patients with stable coronary artery disease: results of the coronary atherosclerosis study measuring effects of rosuvastatin using intravascular ultrasound in Japanese subjects (COSMOS). CARDIOVASC DIABETOL. 2012;11:87 10.1186/1475-2840-11-87 22831708 PMC3444370

[27] Hong LF, Li XL, Guo YL, Luo SH, Zhu CG, Qing P et al Glycosylated hemoglobin A1c as a marker predicting the severity of coronary artery disease and early outcome in patients with stable angina. LIPIDS HEALTH DIS. 2014;13:89 10.1186/1476-511X-13-89 24884794 PMC4070346

[28] Liu T, Li G, Li L, Korantzopoulos P. Association between C-reactive protein and recurrence of atrial fibrillation after successful electrical cardioversion: a meta-analysis. J AM COLL CARDIOL. 2007;49(15):1642–8. 17433956 10.1016/j.jacc.2006.12.042

[29] Zhang Q, Liu T, Ng CY, Li G. Diabetes mellitus and atrial remodeling: mechanisms and potential upstream therapies. CARDIOVASC THER. 2014;32(5):233–41. 10.1111/1755-5922.12089 25065462

